# Telepsychiatry in child and adolescent mental healthcare: clinicians’ perspectives on lessons learned during the COVID-19 pandemic

**DOI:** 10.1007/s00787-026-02995-8

**Published:** 2026-03-03

**Authors:** Vlad-Alexandru Rusu, Robert Vermeiren, Lisa Goossens, Laura Nooteboom

**Affiliations:** Department of Child and Adolescent Psychiatry, LUMC Curium, Leiden University Medical Center, Post Box 15, Leiden, 2300 AA the Netherlands

**Keywords:** Child and adolescent psychiatry, Telepsychiatry, Clinicians, Telehealth, Treatment

## Abstract

Throughout the COVID-19-pandemic, clinicians were forced to immediately adapt their former in-person treatments into remote care for youth with severe psychiatric problems in Child-and-Adolescent Psychiatry (CAP). For the first time, telepsychiatry was implemented on a larger scale than ever before. In this post-COVID-era, as the use of telepsychiatry has shifted from being necessary to elective, it had become increasingly important to understand its advantages and disadvantages. There is an unequivocal call for further research to establish for whom and which types of treatment telepsychiatry may or may not be suitable. Responding to this call, this qualitative study aimed to assess CAP clinicians’ perspectives on the suitability of patient groups and types of treatment for telepsychiatry. Data were collected in five specialised CAP centres in the western and northern provinces of the Netherlands. In total, 288 clinicians participated in an extensive web-based self-report questionnaire and were included in the study. Thematic analysis of the data was performed with ATLAS.ti version 22. The results were divided into five categories: Patients’ disorders, Patients’ characteristics, Patients’ environment, Types of treatment and Characteristics of treatment. Clinicians reported mixed views regarding the suitability of specific disorders or treatments. Specifically, they indicated that patients who were motivated, disciplined and open to telepsychiatry were more likely to be suitable for its use, particularly when presenting with conditions that were mild, stable and less complex. Our findings suggest that telepsychiatry should be approached from a personalized-medicine perspective, taking into account each patient’s individual circumstances and determining suitability in collaboration with patients and their families.

**Supplementary Information:**

The online version contains supplementary material available at 10.1007/s00787-026-02995-8.

## Introduction

Interactions between patients and Child- and Adolescent Psychiatry (CAP) clinicians had changed drastically during the COVID-19 pandemic. Unable to provide their regular, day-to-day care in person, clinicians were forced to immediately adapt their former in-person treatment into remote treatment [1]. As a result, the majority of CAP clinicians rapidly adopted the use of telepsychiatry. Given the broad variety of clinicians implementing telepsychiatry for the first time, it is essential to evaluate and learn from their experiences. This qualitative paper focusses on the experiences of CAP clinicians after a year of employing telepsychiatry during the COVID-19 pandemic in The Netherlands.

Telepsychiatry, defined as providing mental healthcare from a distance through technology, encompasses both direct and indirect (e.g. liaison) contact between patients and clinicians. Common telepsychiatry modalities include phone consultation, video consultation, e-health interventions, and serious gaming [2]. Consequently, both live and pre-recorded material can be part of telepsychiatry. Current literature indicates that telepsychiatry can be effectively improve access to and availability of healthcare [3–8]. Moreover, a growing body of evidence suggests that telepsychiatry is equal to, or in some cases superior to, in-person care in terms of patient outcomes [9] and therapeutic alliance [10–12]. In cases of geographical restrictions, time constraints, or physical disabilities; telepsychiatry is described to be the preferred option [13, 14].

Before the COVID-19 pandemic, the large-scale implementation of telepsychiatry was hindered by clinical, technological and administrative concerns of clinicians; unprepared care systems; and insufficient investment [3, 4, 13, 15, 16]. As a result, the use telepsychiatry was limited to specific patient populations and disorders. Additionally, studies were mostly performed in specific groups of self-selected healthcare providers and patients [17]. During the pandemic, however, telepsychiatry became the primary treatment modality, exposing it to many patients and clinicians that would otherwise never have encountered. Telepsychiatry was implemented on a larger scale than ever before, outside of a research environment and without preparation of patients, clinicians and organizations.

Literature on the adoption of telepsychiatry in CAP during the COVID-19 pandemic suggests both benefits and limitations. While telepsychiatry received widespread acceptance among patients and clinicians [17–20], concerns remain regarding confidentiality, therapeutic alliance, assessment of abuse and neglect, and the ability to interpret non-verbal cues [19–21]. Moreover, recent studies suggest that certain participant groups and types of treatment may be more suitable for telepsychiatry than others [18, 20, 22–29]. Despite some preliminary findings, there is an unequivocal call for further research to establish for whom and for which types of treatment telepsychiatry may or may not be suited.

In the Netherlands, CAP provides multidisciplinary mental healthcare for children and adolescents aged 4–23 years old operating within the second and third line of care. CAP is generally reserved for patients with severe or complex problems that cannot be treated in the first line. Common presentations include (a combination of) internalizing and externalizing disorders, such as depressive disorder, developmental disorders (e.g. autism spectrum disorder (ASD) and attention deficit hyperactivity disorder (ADHD)), anxiety disorders and post-traumatic stress disorder (PTSD). Patients in CAP may receive treatment from any combination of the following experts, including psychiatrists, psychologists, social workers, and psychiatric nurses. Depending on the patient’s individual needs, either ambulatory or in-patient care is available, mostly provided in person. Parents, caretakers, siblings, teachers, and others close to the patient are often involved in both diagnosis and treatment. Whilst telepsychiatry was utilized in CAP before the pandemic, its use was limited to small scale applications, and for specific disorders or treatment modalities.

By studying Dutch CAP clinicians’ experiences with telepsychiatry during the COVID-19 pandemic, this qualitative study aims to provide insights into which patient groups in CAP are best served by telepsychiatry; and which types of treatment CAP clinicians consider most suitable. As telepsychiatry shifts from being necessary to elective, it is increasingly important to understand its advantages and limitations. Such insights can inform further improve the implementation of telepsychiatry in practice.

## Methods

### Context of the study

This study is part of a multicentre research project investigating CAP clinicians’ experiences with telepsychiatry during the COVID-19 pandemic. Data-collection was approved by the Leiden University Medical Centre Ethics Committee, which determined that the study did not require evaluation under the Medical Research Involving Human Subject Act (12-01−2021, N20.058). Data-collection commenced on April 7th, 2021, one year after the start of the COVID measures in the Netherlands, as a post-measurement questionnaire. Data-collection was achieved through CASTOR, an online platform for secure data capture and management.

### Participants

Data were collected in five specialised CAP centres in the western and northern provinces of the Netherlands. All healthcare personnel (*n* = 3597) employed by the participating centres received an e-mail invitation to partake in the study; however, not all personnel were deemed eligible for inclusion as a large number did not provide patient care. To be included in the study, participants were required to (1) be practicing child- and adolescent mental healthcare clinicians, (2) have provided telepsychiatry during the pandemic, and (3) have answered at least one of the open-ended questions in the questionnaire attached to the e-mail invitation.

A total of 289 clinicians met the inclusion criteria and gave informed consent to participate in the study. One participant was later excluded because their responses consisted of nonsensical entries (i.e., “x” or “?”). In total, 288 clinicians were included in the study.

### Measures

A web-based self-report questionnaire was specifically developed by the authors to study CAP clinicians’ experiences with telepsychiatry during the COVID-19 pandemic. The content of the questionnaire was designed based on current literature on telepsychiatry and was later revised to include open-ended questions to allow for more in-depth exploration (post-measurement questionnaire).

For this study, we focused on these open-ended questions regarding the suitability of telepsychiatry for distinct patient groups and treatment modalities. These included: (1) For which patients is telepsychiatry suitable? (2) For which patients is telepsychiatry not suitable? (3) For which treatment modalities is telepsychiatry suitable? (4) For which treatment modalities is telepsychiatry not suitable?

### Data analysis

Data relevant to the study were loaded into ATLAS.ti version 22. To improve the authors’ familiarity with the data, an initial scan of the raw data was performed. As a built-in quality and reproducibility check, data were coded separately by both V.R. and L.G.. Excerpts that were coded identically remained unchanged, whereas excerpts that were coded differently were discussed until a consensus was reached. Data were coded manually and inductively under L.N.’s supervision as an experienced qualitative researcher.

Subsequently, data were screened for topics or issues that could be used as central organizing concepts for themes and sub-themes, defined as patterns of meaning within the data, and themes within themes [30]. Then, categories were thematically analysed from the dual vantage points of suitability and non-suitability for telepsychiatry. Observed patterns in the themes were clarified through in-depth analysis of the answers to the open-ended questions as provided by the clinicians.

## Results

### Participants

A total of 288 clinicians were included in the study from five CAP institutions. For this study, professions were grouped into four broad categories according to the Dutch model of mental healthcare. Categories were defined based on the required level of education; work activities; and the “BIG-register”, a national registry for healthcare professionals in the Netherlands. Professions of the participants included psychiatrists, medical residents, clinical-psychologists, and psychotherapists (14.2%); healthcare-psychologists and general remedial educationalists (22.2%); systemic therapists, remedial educationalists, and psychologists (34.7%); and social workers, district team workers, child and family centre workers, and psychiatric nurses (22.9%).

Participants diagnosed and treated patients under 12 years of age (43.3%), over 12 years of age (76.4%) and parents (41.0%).

### Thematic analysis

Thematic analysis resulted in five categories and 33 themes. The categories are: (1) Patients ‘disorders, (2) Patients’ characteristics, and (3) Patients’ environment (including therapeutic alliance); and two categories regarding which treatment modalities are most suitable for telepsychiatry: (4) Types of treatment, and (5) Characteristics of treatment. In the following section, the findings are described per category. Quotes, translated from Dutch to English by the authors (V.R., L.N.), therefore considered paraphrased, were included to further illustrate CAP clinicians’ impressions of telepsychiatry. An overview of the categories and themes is provided in Table 1 and a detailed description of the codes, themes and categories can be found in Appendix A.

**Table 1:** Categories and themes

**Patients’ Disorders**	**Patients’ characteristics**	**Patients’** **Environments**	**Types of treatment**	**Characteristics of treatment**
Neurodevelopmental disorders	Communication & interpersonal skills	Environmental factors	Informative meetings & scheduling	Method
Anxiety & mood disorders	Stability & severity of problems	Therapeutic relationship	Intake & diagnosis	Complexity
Personality disorders	Motivation & self-awareness	Familial factors	Evaluation & advise	Frequency
Feeding & eating disorders	Behaviour & coping	Available tools	(Psycho-)education	Group size
Psychotic disorders	Demographical characteristics		Psychotherapy	
Trauma- & stressor-related disorders			Behavioural therapy	
Suicidality & acute disorders			Exposure therapy	
Substance-related & addictive disorders			Occupational therapy	
			System therapy	
			Supportive therapy	
			Imagination therapy	
			Therapy for specific disorders; not otherwise specified	
			(communication-) Training	
			Peer consultation	
			Mindfulness	
			Medication consultation	

#### Patients’ disorders

The category “Patients’ disorders” contains a description of disorders mentioned by CAP clinicians regarding the questions: “For which patients is telepsychiatry suitable” and “For which patients is telepsychiatry not suitable”. Clinicians reported a variety of disorders, such as neurodevelopmental disorders, personality disorders and anxiety- and mood disorders, and described them as being both suitable and less suitable to be treated via telepsychiatry. Specifically, clinicians’ reports indicated that it was generally not the disorder that was deemed suitable or less suitable for telepsychiatry, but rather its manifestation in practice. Severity, complexity, and stability were described as the most important factors in (un)suitability of a condition for telepsychiatry treatment. Clinicians reported that the more severe, complex, or unstable a condition, the less suitable it was for telepsychiatry. Correspondingly, mild, stable, and less complex conditions were regarded as highly suitable for telepsychiatry.

*“What I can say is that I think that the more serious the disorder is*,* the more difficult it is to apply telepsychiatry.” P1*.

In line with the aforementioned, the subcategory “suicidality & acute disorders” was subject to an almost unanimous view. While there was some (albeit very little) disagreement regarding suicidality; patients showing any form of crisis or auto-mutilation were collectively seen as less suitable for telepsychiatry. Other disorders that were consistently mentioned as being less suitable were “(mild) intellectual disability”, and “selective mutism”. Although the subcategory “substance-related & addictive disorders” was also unanimously seen as less suitable for telepsychiatry, only a single clinician referred to any disorder befitting this subcategory.

*“Although telepsychiatry is most suited for patients that are already on the right track regarding treatment and therapeutic relationship; telepsychiatry can also be applied as a way to keep your finger on the pulse when it comes to crisis-sensitive patients.” P2*.

#### Patients’ characteristics

The category “Patients’ characteristics” contains the characteristics that were reported by clinicians when they described for which patients they deemed telepsychiatry to be suitable and less suitable. Clinicians highlighted the importance of patients’ communication skills and their ability to comfortably express themselves through telepsychiatry. While patients with little to no mastery of the Dutch language were deemed less suitable by definition; clinicians were adamant regarding the importance of verbal communication. To prevent miscommunications and/or missed information, the patient must be able to reliably convey their thoughts and feelings. Likewise, the patient must be able to understand the clinician. As non-verbal communication is often less noticeable or completely out of view, clinicians reported to only be able to consistently rely on verbal communication when providing telepsychiatry. As a result, patients heavily relying on non-verbal communication were deemed less suitable.

*“It is noticeable that if you’re having a difficult conversation*,* or the patient is experiencing anxiety*,* it is easier for them say less or stop the conversation altogether. Moreover*,* if patients are closed-off or speak with few words*,* it is difficult to not be able to rely on their body language to see how it’s really going.” P3*.

Clinicians reported unmotivated, undisciplined, or highly impulsive patients as less suitable for telepsychiatry, as they were often found to be distracted, or simply doing something else during online therapy. Conversely, motivated, disciplined, and self-regulating patients were seen as highly suitable. An often-overlooked facet of motivation was patients’ openness to telepsychiatry. Clinicians emphasized that ignoring patient preferences could negatively influence treatment outcomes.

*“Patients reluctant to using telepsychiatry should definitely be seen face-to-face.” P1*.

Self-reflection, initiative, and being able to set boundaries were seen as behavioural traits that improved patients’ suitability for telepsychiatry. Moreover, a flexible attitude and having no resistance towards the treatment trajectory were said to be vital. On the other hand, shyness, closedness, and more withdrawn behaviour was said to be detrimental to telepsychiatry. Identified as downright less suitable traits were restlessness, evasiveness, and distrustfulness.

An at least an average IQ and basic digital skills were identified as prerequisites for telepsychiatry. Clinicians noted that patients lacking the capacity to understand or use telepsychiatry modalities would be better served with face-to-face care. There was no consensus regarding exact cut-off points for the minimal age or IQ scores to be able to take part in telepsychiatry; instead, clinicians recommended a personalized approach. In addition, patients needed to have the financial means to afford the hardware required for telepsychiatry. Patients with a lower socio-economical-status were said to be at a clear disadvantage.

*“Unsuitable for telepsychiatry are patients that are unable to manage the technical aspects of video-calls*,* for example some patients with a Mild Intellectual Disorder (MID).” P4*.

#### Environment & therapeutic alliance

The category “Environment and therapeutic alliance” consists of clinicians’ descriptions of environmental factors and relational dynamics influencing the suitability of telepsychiatry. Clinicians agreed that a safe and private environment is essential: a quiet, peaceful environment where the patient feels at ease and can comfortably speak as their privacy is respected. Patients with unsafe or crowded home environments were identified as less suitable for telepsychiatry. On the other hand, telepsychiatry was seen as ideal for patients requiring more flexible scheduling, or patients with geographical or physical restrictions. Proper hardware and a stable internet connection were identified as prerequisites for telepsychiatry. A shortcoming herein, on either end (patient or clinician), was said to be particularly detrimental. Sound delays, audio glitches, and other technical issues were said to be immensely disruptive.

*“I’ve noticed that preconditions are central to the application of telepsychiatry. In my opinion*,* poor (internet) connectivity or too little privacy to conduct treatment hinders the use of telepsychiatry.” P5*.

Some clinicians reported difficulties establishing therapeutic alliances with patients through telepsychiatry, this applied to both new patients and patients without an already flourishing therapeutic alliance. On the other hand, clinicians specifically described patients with an already well-established therapeutic alliance as suitable for telepsychiatry. Although clinicians noted that interactions between family members were less visible during telepsychiatry than in face-to-face settings, it was seen as ideal for some families as members were able to join from different locations. Moreover, clinicians mentioned the unique benefit of being able to have a look into the patient’s home situation.

*“Telepsychiatry makes it easier to involve (both) parents as it makes it possible to connect with them*,* even if they are unable to physically attend an appointment. Moreover*,* for some parents it helps that they do not have to be in the same room with each other.” P6*.

#### Types of treatment

The category “Types of treatment” consists of clinicians’ reports on the questions: “For which types of treatment is telepsychiatry suitable” and “For which types of treatment is telepsychiatry not suitable”. Most types of treatment were seen as both suitable and less suitable for telepsychiatry, as the opinions of clinicians varied per treatment type. However, the subthemes “peer consultation” and “informative meetings & scheduling*”* were unanimously considered suitable for telepsychiatry. Moreover, forms of “supportive therapy” and “coaching in daily functioning” were almost unanimously considered suitable. On the other hand, all forms of “occupational therapy” were reported as less suitable, often attributed to the incompatibility of physical materials with telepsychiatry.

*“Unsuitable interventions through telepsychiatry are observations and interventions that require physical materials or exercises.” P7*.

#### Characteristics of treatment

This category consists of clinicians’ reports regarding the characteristics of treatment in relation to telepsychiatry. As with the category “patients’ disorders”, clinicians’ reports in this category indicated that it was often not the type of treatment that was deemed suitable or less suitable, but rather its characteristics. Clinicians reported preferring to see the patient in-person during complex, intensive and emotionally charged treatments (e.g. trauma treatment or crisis interventions). On the other hand, clinicians had no reservations regarding simple, more straightforward therapy. To cover telepsychiatry’s shortcomings, many clinicians reported being proponents of blended therapy (telepsychiatry combined with face-to-face treatment).

*“When it comes to emotional conversations such as test results or diagnoses*,* I prefer having physical appointments so that I am better able to grasp patients’ and parents’ emotions.” P7*.

Structured and protocolled methods were reported to be highly suitable for telepsychiatry. On the contrary, unstructured, and non-protocolled methods were reported to be less suitable. While questionnaires and digital materials were said to be effectively employable in telepsychiatry, physical materials, and other psychoanalytical- and psychotherapy tools were said to be incompatible. Furthermore, any physical components of therapy (i.e., examinations and exercises) were seen as unemployable. Another treatment characteristic said to be important to take into consideration was “group size”. While group therapy was a more controversial topic, individual therapy was unanimously seen as suitable for telepsychiatry.

*“Interventions with clear instructions and less emphasis on therapeutic alliance are most suitable for telepsychiatry.” P8*.

## Discussion

Telepsychiatry has been an invaluable tool during the COVID-19 pandemic in facilitating the continuation of care in CAP and appears to retain much of its popularity in the post-COVID-19 era [31]. Answering the unequivocal call for further research [18, 20, 22–29], our study aimed to assess CAP clinicians’ perspectives on the suitability of patient groups and types of treatment for telepsychiatry.

### Patients

The findings of this study indicate that, as long as certain criteria are met and the patient is open to telepsychiatry, telepsychiatry can be employed for a broad range of patient groups. In line with previous literature [28]; the severity, complexity and stability of patients’ disorders provide a strong indicator of patients’ suitability for telepsychiatry. The more severe, complex, or unstable the patient’s condition, the less suitable they are for telepsychiatry and vice versa. Similarly, another indicator of suitability was linked to personal characteristics and capabilities. Adequate communication, IQ, age, motivation, discipline, self-regulation, initiative, openness, and digital knowledge were described as predictors for patients’ suitability for telepsychiatry. Correspondingly, shortcomings in these characteristics were seen as strong indicators of reduced suitability for telepsychiatry.

Not to be underestimated is the importance of a safe and stable environment, to be interpreted as a quiet and private environment where the patient could comfortably speak during telepsychiatry. Moreover, consistent with earlier studies [28, 29], the availability of proper hardware and a stable internet connection were deemed pre-requisites to safeguard the quality of therapy.

In conclusion, to provide personalized care [32] it is recommended that practitioners assess the patient alongside the aforementioned characteristics and come to a shared decision, with both patient and parents, of whether or not telepsychiatry is suitable for the individual in question.

### Types of treatment

The findings of this study indicate that telepsychiatry can be employed for a broad range of treatment modalities. Telepsychiatry was deemed most suitable for ongoing, simple, and straightforward therapy (e.g. structured and protocolled methods and psychoeducation), and less suitable for intensive, complex or emotionally charged therapy (e.g. trauma therapy or crisis intervention). A pre-established therapeutic alliance was reported to be profoundly more important than the treatment type itself, a finding that aligns with existing literature [22, 29].

Our findings suggest that, when possible, it is highly beneficial for clinicians in CAP to invest in building a therapeutic relationship in person before implementing telepsychiatry. Moreover, clinicians must consider the complexity of treatment and physical components that may be incompatible with telepsychiatry. However, for clinicians to deliver personalized telepsychiatry to a vulnerable patient group, proper training and education on telepsychiatry, even early on during the course of medicine, is crucial [33, 34]. Additionally, when studying the actual effects of telepsychiatry, not only patient characteristics, but also characteristics of the clinician (e.g. clinical experience, education and technology literacy) and organizational readiness should be considered.

### Strengths and limitations

The entire research project was set up at the beginning of the COVID-19 pandemic, enabling the authors to distribute the first questionnaire only two weeks into the first lockdown in the Netherlands. The aforementioned allowed for unique insights into clinicians’ first experiences with telepsychiatry; however, it simultaneously resulted in limited, indirect interactions (questionnaires). Therefore, we added open ended questions to the follow up measurement on which this study is based, to gain a more in-depth view of patient populations suitable and less suitable for telepsychiatry. However, interview-based studies following up on the findings of this paper are strongly encouraged, as are studies aiming to better understand the perspectives and expectations of clinicians, but also patients and their parents regarding telepsychiatry.

An important strength of this study is its sheer size and diversified recruitment from different units of care. The broad distribution of the questionnaire allowed for a firm study population of Dutch CAP clinicians. On the other hand, personnel that did not provide direct care for patients may have received the questionnaire, resulting in a seemingly lower response rate. Moreover, it is unknown whether the study population as a whole was impartial or leaned towards either a critical or a positive view of telepsychiatry. Also, the study was solely conducted in the Netherlands, a high-income country, so findings cannot be generalized to other, especially low-income countries without caution. To conclude, we do not have information on the actual effect of telepsychiatry on patients’ wellbeing, as this study is based on clinicians’ experiences. Also, we did not take clinician related variables in our analysis, such as technology literacy and clinical experience. Therefore, it is strongly recommended that future studies integrate both direct treatment outcomes and clinician characteristics more strongly in their designs.

## Conclusion

In conclusion, our findings suggest that clinicians working in CAP view telepsychiatry as a useful tool for most patients, though not in all circumstances. Specifically, clinicians reported that patients who were motivated, disciplined and open to telepsychiatry seemed more likely to be suitable for its use, especially when presenting with conditions that were mild, stable and less complex. Attention was also drawn to the importance of practical considerations such as a suitable home environment and stable connectivity. Our findings suggest that telepsychiatry should be approached from a personalized-medicine perspective, considering each patient’s individual circumstance and determine the suitability in conjunction with patients and their families.

## Supplementary Information

Below is the link to the electronic supplementary material.

Supplementary Material 1 (DOCX 17.1 KB)

## Data Availability

Restricted access to the data, please contact the corresponding author for more information.
