# Supplementary material for: Telepsychiatry in child and adolescent mental healthcare: clinicians’ perspectives on lessons learned during the COVID-19 pandemic
Source: Eur Child Adolesc Psychiatry. 2026 Mar 3;35(6):1883–90. doi: 10.1007/s00787-026-02995-8 (PMC13337745; doi:10.1007/s00787-026-02995-8)
Supplement: Supplementary file 1 — Supplementary Material 1 (DOCX 17.1 KB) [file 787_2026_2995_MOESM1_ESM.docx]

| **Appendix A. Overview of categories and themes** |  |  |
| --- | --- | --- |
|  | **Suitable or beneficial for telepsychiatry** | **Less suitable for Telepsychiatry** |
| **Patients’ Disorders** |  | Suicidality & Acute disorders |
|  |  | (Mild) intellectual disabilities |
|  |  | Selective mutism |
|  | Mild conditions | Severe conditions |
|  | Simple conditions | Complex conditions |
|  | Stable conditions | Unstable conditions |
|  | Conditions not requiring a physical examination | Conditions requiring a physical examination |
| **Patients’ Characteristics** |  | Patients heavily reliant on non-verbal communication |
|  | Patients adequately skilled in (verbal) communication | Patients inadequately skilled in (verbal) communication |
|  | Motivated patients | Unmotivated patients |
|  | Disciplined patients | Undisciplined patients |
|  | Self-regulating patients | Highly impulsive patients |
|  | Patients with a preference for telepsychiatry | Patients with a preference for face-to-face care |
|  | Patients capable of self-reflection |  |
|  | Patients showing initiative |  |
|  | Patients able to set boundaries |  |
|  | Patients with a flexible attitude |  |
|  | Patients with little to no resistance towards treatment trajectory |  |
|  |  | Patients that are shy |
|  |  | Patients that are closed off |
|  |  | Patients that are withdrawn |
|  |  | Patients that are restless |
|  |  | Patients that are evasive |
|  |  | Patients that are distrustful |
|  | Patients with an at least average IQ | Patients with a lower than average IQ |
|  | Patients of adequate age | Patients that are too young to comprehend or utilize telepsychiatry modalities |
|  | Patients with adequate digital skills | Patients with inadequate digital skills |
| **Patients’ environments** | Safe environments where privacy is respected | Unsafe or crowded environments |
|  | Patients requiring flexible scheduling |  |
|  | Patients with factors impeding them in being physically present |  |
|  | Patients with an already established therapeutic alliance | New patients or patients without an adequate therapeutic alliance |
|  | Family members are able to log in from different locations | Interactions between family members are lost during telepsychiatry |
|  | The clinician is able to have a look into the patient’s home situation |  |
|  | Proper hardware and a stable internet connection | Lack of proper hardware and a stable internet connection |
| **Types of treatment** | Informative meetings & scheduling |  |
|  | Supportive therapy |  |
|  |  | All forms of occupational therapy |
| **Characteristics of treatment** | Structured methods | Unstructured methods |
|  | Protocolled methods | Non-protocolled methods |
|  | Questionnaires, and digital material | Other psychoanalytical and psychotherapy tools, and physical material |
|  | Simple, straightforward treatment methods | Complex treatment methods |
|  |  | Emotionally charged treatment methods |
|  | Ongoing treatment trajectories |  |
|  | Singular treatments |  |
|  |  | Intensive treatment trajectories |
|  | Individual therapy |  |
|  | Blended therapy |  |
|  | “To keep a finger on the pulse” contact |  |
